# Immunogenicity of Enhanced Influenza Vaccines Against Mismatched Influenza Strains in Older Adults: A Review of Randomized Controlled Trials

**DOI:** 10.1111/irv.13286

**Published:** 2024-04-09

**Authors:** John Youhanna, Vy Tran, Randall Hyer, Alexander Domnich

**Affiliations:** ^1^ CSL Seqirus Ltd Summit New Jersey USA; ^2^ Baruch S. Blumberg Institute Doylestown Pennsylvania USA; ^3^ Hygiene Unit San Martino Policlinico Hospital Genova Italy

**Keywords:** adjuvanted influenza vaccine, heterologous influenza viruses, high‐dose influenza vaccine, influenza vaccine immunogenicity, older adults, recombinant influenza vaccine

## Abstract

Antigenic drift is a major driver of viral evolution and a primary reason why influenza vaccines must be reformulated annually. Mismatch between vaccine and circulating viral strains negatively affects vaccine effectiveness and often contributes to higher rates of influenza‐related hospitalizations and deaths, particularly in years dominated by A(H3N2). Several countries recommend enhanced influenza vaccines for older adults, who are at the highest risk of severe influenza complications and mortality. The immunogenicity of enhanced vaccines against heterologous A(H3N2) strains has been examined in nine studies to date. In six studies, an enhanced, licensed MF59‐adjuvanted trivalent inactivated influenza vaccine (aIIV3) consistently increased heterologous antibody titers relative to standard influenza vaccine, with evidence of a broad heterologous immune response across multiple genetic clades. In one study, licensed high‐dose trivalent inactivated influenza vaccine (HD‐IIV3) also induced higher heterologous antibody titers than standard influenza vaccine. In a study comparing a higher dose licensed quadrivalent recombinant influenza vaccine (RIV4) with HD‐IIV3 and aIIV3, no significant differences in antibody titers against a heterologous strain were observed, although seroconversion rates were higher with RIV4 versus comparators. With the unmet medical need for improved influenza vaccines, the paucity of studies especially with enhanced vaccines covering mismatched strains highlights a need for further investigation of cross‐protection in older adults.

## 1 Introduction

Seasonal influenza carries a significant burden, causing up to 650,000 deaths annually across the globe as well as billions of dollars' worth of direct and indirect costs to individuals and society [1, 2]. Rapid viral evolution lies behind the toll of influenza as new strains capable of evading the human immune response continually arise due to antigenic drift, in which selection pressure promotes mutations in the viral surface antigens hemagglutinin (HA) and neuraminidase (NA) [3, 4]. Consequently, the World Health Organization (WHO) annually reviews epidemiologic patterns and vaccine effectiveness, determines which circulating influenza viruses are most likely to predominate in the coming season, and recommends those strains for inclusion in the next year's influenza vaccine. However, midseason antigenic drift and other factors such as antigenic changes that occur in vaccine viruses grown in chicken eggs (i.e., egg adaptation) frequently reduce vaccine effectiveness, as circulating strains no longer match the viruses contained within seasonal influenza vaccines [5].

Of the four virus types included in seasonal influenza vaccines—A(H1N1), A(H3N2), B/Victoria, and B/Yamagata—A(H3N2) is most often associated with reduced vaccine effectiveness. In addition, waning immunity in vaccinated individuals over the course of an influenza season has been documented more often with A(H3N2) than with other virus types [6, 7, 8, 9]. Both antigenic drift and egg adaptation are more likely to occur with A(H3N2) than other viral types, and influenza seasons predominated by A(H3N2) are often characterized by reduced vaccine effectiveness driven by mismatch between circulating and vaccine viruses [10, 11, 12].

Rates of influenza‐related hospitalizations and deaths also tend to be higher in mismatched seasons, even when vaccine effectiveness is relatively high [13, 14, 15, 16, 17, 18, 19, 20, 21, 22, 23, 24, 25]. Older adults are particularly vulnerable to these outcomes. This group is more susceptible to influenza infection due to immunosenescence or age‐related changes in immune system responses to infection [26]. Waning immunity may also be more common in older versus younger adults [6, 9, 27]. In addition, the highest rates of influenza‐related deaths and hospitalizations occur among adults ≥ 65 years of age, whose risk of influenza complications is increased owing to a higher frequency of comorbidities and frailty [26, 28].

To improve influenza protection for older adults, the WHO, Advisory Committee on Immunization Practices (ACIP), and other agencies recommend enhanced influenza vaccines for older adults [5, 9]. The recommended licensed vaccines include MF59‐adjuvanted inactivated influenza vaccine (aIIV; Fluad or Fluad Quadrivalent; CSL Seqirus Ltd. Parkville, Australia), high‐dose inactivated influenza vaccine (HD‐IIV; Fluzone High Dose and Fluzone High Dose Quadrivalent, Sanofi, Paris, France), and a (higher than standard dose) quadrivalent recombinant influenza vaccine (RIV4; FluBlok Quadrivalent, Sanofi, Paris, France). The higher‐dose vaccines contain either 60 μg HA per strain (HD‐IIV) or 45 μg HA per strain (RIV4), which increases the magnitude of the immune response compared with standard influenza vaccines containing 15 μg HA per strain [29]. aIIV contains the standard dose of antigen—15 μg HA per strain—enhanced with the MF59 adjuvant, a squalene‐based, oil‐in‐water emulsion, which increases not only the magnitude but also the breadth of the immune response by enhancing production of cross‐reactive antibodies [30]. The development of cross‐reactive neutralizing antibodies, referred to as cross‐protection is particularly important in mismatched seasons.

Clinical trials and observational studies have generally shown that, compared with standard influenza vaccines, enhanced vaccines improve protection from influenza infection and/or medical visits, hospitalizations, and deaths in older adults [9, 31, 32, 33, 34, 35, 36, 37, 38, 39, 40, 41, 42, 43, 44, 45, 46]. Few studies, however, have examined the effects of these vaccines during mismatched seasons, and even fewer have examined immune responses to circulating virus strains that differ antigenically from the corresponding vaccine virus. This may include heterologous strains, which are generally defined as strains associated with an ≥ 8‐fold reduction in immune responses measured with the hemagglutination inhibition (HI) assay, which is equivalent to a difference of three antigenic units (or the distance between viruses in the antigenic map) [47]. Here, we review available data on this important aspect of protection from influenza with a focus on immunogenicity studies involving heterologous, drifted, or mismatched influenza strains.

## 2 Methodology

**FIGURE 1:**
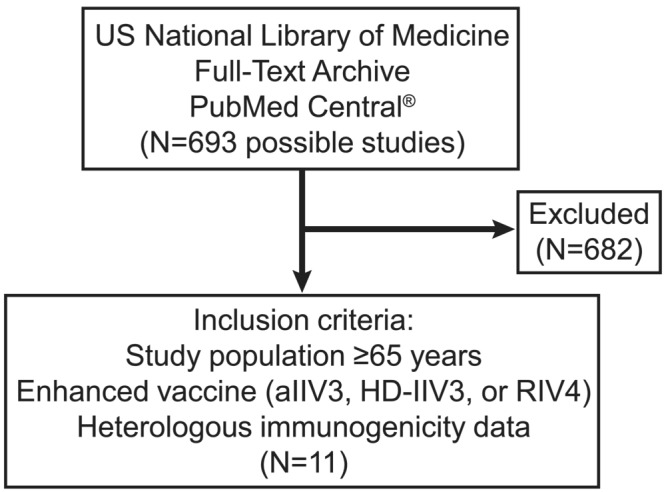
Results of literature search used to identify eligible randomized, clinical trials reporting immunogenicity data for enhanced vaccines tested against heterologous, drifted, or mismatched strains.

This review was designed as a narrative review of available data and is confined to information available in the published references described herein. We reviewed databases of the US National Library of Medicine full‐text archive, PubMed Central, to identify randomized clinical trials with study designs meeting the following inclusion criteria: immunogenicity data for enhanced vaccines tested against heterologous, drifted, or mismatched strains and a study population aged ≥ 65 years. Initial search filters comprised the terms “clinical trial” and “randomized controlled trial” with a date range of “2014 to present” (search date: May 31, 2023). Subsequently, the following search strings was applied: (((((((Fluzone‐HD) OR (HD‐TIV)) OR (HD‐QIV)) OR (Efluelda)) OR (QIV‐HD)) OR (TIV‐HD) AND (mismatch)) OR (drifted strain)). Additional search strings included the terms “aTIV,” “aQIV,” or “RIV4” in place of terms related to HD‐IIV3, with other parameters the same. Studies involving adults younger than 65 years or pediatric populations were excluded. Out of 689 possible studies, we identified 10 studies with immunogenicity data plus one meta‐analysis (Figure 1) [32, 39, 40, 48, 49, 50, 51, 52, 53, 54, 55].

Table 1 summarizes the study designs, main or primary endpoints, strains analyzed in each study, and antigenic difference methodology and results as described in each study [32, 39, 40, 48, 49, 50, 51, 52, 53, 54, 55, 56, 57, 58, 60, 61, 62, 63, 64, 65, 66].

**TABLE 1:** Designs of studies evaluating vaccine‐induced antibody responses to heterologous influenza virus strains.

Study, season(s)	Design	Main or primary endpoint(s)	Heterologous strain analysis		
			Strains tested	Antigenic difference, Ref/Hom/Vaccine and Het strains	Antigenic analysis method
De Donato et al. [48], 1993–1994, 1994–1995, 1995–1996	RCT, *N* = 211 Age: 64–87 years Study vaccines: aIIV3, IIV3‐sub1	HI GMT % subjects with ≥ 4‐fold increase in HI titer versus baseline and with HI titer ≥ 128	Hom: A/Beijing/32/92 Het: A/Shandong/9/93	Not described	NT titer, MDCK cell culture [56]
Del Giudice et al. [49], 2003–2004	RCT, *N* = 119 Age: 61–91 years Study vaccines: aIIV3, IIV3‐sub1, IIV3‐split	HI GMT Seroprotection	Hom: A/Panamal2007/99 Het: A/Wyoming/3/2003^a^	Not described	HI titer [57]
Ansaldi et al. [40], 2004–2005	RCT, *N* = 50 Age: ≥ 65 years Study vaccines: aIIV3, IIV3‐sub1	HI GMT HI seroprotection rate HI seroconversion rate NT GMT NT MFI	Hom: A/Wyoming/3/03		HI titer, ferret sera [58]
			Het:		
			A/Panama/2007/99	8‐ to 16‐fold	
			A/California/7/04	8‐fold	
			A/Wisconsin/67/05	16‐fold	
Ansaldi et al. [39], 2004–2005 – 2006–2007	RCT, *N* = 50 Age: ≥ 65 years Study vaccines: aIIV3, IIV3‐sub1	HI GMT HI MFI HI seroconversion rate HI seroprotection rate MN GMT MN seroconversion rate MN titer ≥ 1:40	Ref, egg‐grown: A/California/7/04		HI titer, ferret sera [58]
			Hom, cell‐grown:		
			A/Genoa/59/04	4‐fold	
			A/Genoa/02/05	None	
			A/Genoa/11/05	None	
			A/Genoa/47/05	2‐fold	
			A/Genoa/62/05	2‐fold	
			Het, egg‐grown:		
			A/Wyoming/3/03	2‐fold	
			A/Wisconsin/67/05	4‐fold	
			A/Brisbane/10/07	16‐fold	
			Het, cell‐grown:		
			A/Genoa/13/04	4‐fold	
			A/Genoa/27/04	4‐fold	
			A/Genoa/47/04	4‐fold	
			Het, drifted:		
			A/Genoa/02/07	128‐fold	
			A/Genoa/03/06	64‐fold	
			A/Genoa/03/07	256‐fold	
Baldo et al. [50], 1998–1999 (RCT conducted), 2006–2007 (sera re‐tested)	Re‐test of sera collected during a previous RCT [59], *N* = 199 Age: ≥ 65 years Study vaccines: aIIV3, SVV, IIV3‐split2	HI GMT HI MFI % subjects with ≥ 4‐fold increase in HI titer	Hom: A/Beijing/262/95 (H1N1), A/Sydney/5/97 (H3N2), Beijing/184/93 (B strain) Het: A/New Caledonia/20/99 (H1N1), A/Wisconsin/67/2005 (H3N2), Malaysia/2506/2004 (B strain)	Not specified; phylogenetic trees provided in Figure 1 of original article	NIAID IRD data [60]
Song et al. [51], 2009–2010	RCT, *N* = 95 Age: ≥ 65 years Study vaccines: aIIV3, IIV3‐split3	HI GMT HI seroprotection rate HI seroconversion rate	Hom: A/Brisbane/59/2007 (H1N1), A/Brisbane/10/2007 (H3N2) Het: A/California/7/2009 (H1N1), A/New Caledonia/20/1999 (H1N1), A/Solomon Island/3/2006 (H1N1), A/Wisconsin/67/2005 (H3N2)	Not specified	HI titer, turkey erythrocytes [61, 62]
Camilloni et al. [52], 2011–2012	RCT, *N* = 80 Age: ≥ 64 years Study vaccines: aIIV3, IIV3‐split4 (intradermal)	No. of subjects with HI ≥ 40 HI GMT HI MFI Seroconversion rate	Hom (H3N2): A/Perth/16/09 Het (H3N2): A/Perugia/06/12, A/Perugia/20/12, A/Perugia/44/12, A/Perugia/50/12	Not specified; phylogenetic trees provided in Figure 1 of original article	HI titer, turkey erythrocytes [63]
Frey et al. [32], 2010–2011	RCT, *N* = 7802 Age: ≥ 65 years Study vaccines: aIIV3, IIV3‐sub2	HI GMT^a^ HI seroconversion rate	Vaccine: A/Perth/16/2009 (H3N2), B/Brisbane/60/2008 Het: A/Brisbane/10/2007 (H3N2), A/Wisconsin/67/2005 (H3N2), B/Malaysia/2506/2004	> 4–8 fold difference between vaccine and Het strains	HI titer, ferret sera [64]
Dunning et al. [53], 2011–2012, 2012–2013	RCT, *N* = 675 Age: ≥ 65 years Study vaccines: HD‐IIV3, IIV3	HI GMT Correlate of protection	Ref: A/Victoria/361/2011 (egg‐adapted vaccine strain)^b^ Het: A/Victoria/361/2011 (circulating strain)^b^	4 AA substitutions	CDC data [65]
Belongia et al. [55], 2017–2018	RCT, *N* = 89 Age: 65–74 years Study vaccines: aIIV3, HD‐IIV3, RIV4	Primary: MN GMT, MN GMFR Secondary: MN seroconversion rate	Hom, cell‐grown: A/Hong Kong/4801/2014 Het: A/Singapore/INFIMH‐16–0019/2016, A/Kentucky/29/2017, A/Kansas/14/2017	Not specified	CDC data [66]

Abbreviations: AA, amino acid; aIIV3, MF59‐adjuvanted trivalent influenza vaccine (Fluad; CSL Seqirus Ltd. Parkville, Australia); CBER, Center for Biologics Evaluation and Research; CHMP, European Committee for Medicinal Products for Human Use; GMFR, geometric mean fold rise; GMT, geometric mean titer; HD‐IIV3, high‐dose trivalent influenza vaccine (Fluzone High Dose, Sanofi, Paris, France); Het, heterologous strain; HI, hemagglutination inhibition; Hom, homologous strain; IIV3, nonadjuvanted trivalent influenza vaccine (brand not specified); IIV3‐split1, nonadjuvanted split virion trivalent influenza vaccine (Begrivac; Novartis Vaccines, Basel, Switzerland); IIV3‐split2, nonadjuvanted split virion trivalent influenza vaccine (Mutagrip, Pasteur Merieux MSD, Lyon France); IIV3‐split3, nonadjuvanted split virion trivalent influenza vaccine (GCFlu, Green Cross, Seoul, Korea); IIV3‐split4, nonadjuvanted split virion, intradermal trivalent influenza vaccine (Intanza, Sanofi‐Pasteur, Lyon, France); IIV3‐sub1, nonadjuvanted subunit trivalent influenza vaccine (Agrippal, Novartis Vaccines, Basel, Switzerland); IIV3‐sub2, nonadjuvanted subunit trivalent influenza vaccine (Agriflu, Novartis Vaccines, Basel, Switzerland); IIV4, nonadjuvanted quadrivalent influenza vaccine (FluQuadri, Sanofi Pasteur, Lyon, France); MDCK, Madin‐Darby canine kidney; MFI, mean fold increase; MN, microneutralization; NIAID IRD, National Institute of Allergy and Infectious Diseases‐funded Influenza Research Database; RCT, randomized controlled trial; Ref, reference strain; RIV4, quadrivalent recombinant influenza vaccine (FluBlok Quadrivalent, Sanofi, Paris, France); SVV, trivalent subunit virosomal vaccine (InflexalV, Swiss Serum and Vaccine Institute, Bern, Switzerland).

^a^ Co‐primary objectives: (1) establish the immunological equivalence of three consecutive lots of aIIV3 for all three homologous strains; (2) demonstrate noninferiority (against all 3 strains) and superiority (in at least two of three strains) of postvaccination GMTs and seroconversion rates of aIIV3 compared with IIV3 based on the CBER criteria; and (3) evaluate the immunogenicity of aIIV3 according to the CHMP criteria against the 3 strains.

^b^ Seasonal mismatch due to egg adaptation of A/Victoria/361/2011 reference virus, not antigenic drift of circulating A/Victoria/361/2011.

## 3 Enhanced Influenza Vaccine Immunogenicity Against Drifted A(H3N2) and Other Heterologous Strains

Ten studies, including a meta‐analysis, describe the immunogenicity of the licensed MF59‐adjuvanted trivalent influenza vaccine (aIIV3; Fluad) against heterologous strains, primarily A(H3N2) variants (Table 2) [32, 39, 40, 48, 49, 50, 51, 52, 54, 55]. One of the 10 studies compared aIIV3 with non‐adjuvanted HD‐IIV3 and RIV4 [55]. The 11th study compared HD‐IIV3 with standard‐dose, nonadjuvanted IIV3 [53].

**TABLE 2:** Results of studies evaluating vaccine‐induced antibody responses to heterologous influenza virus strains.

Study/design	Immunogenicity outcome			
	Vaccine, Ref, or Hom influenza strain(s)		Het influenza strain(s)	
	Primary vaccine^a^	Comparator	Primary vaccine^a^	Comparator
De Donato et al. [48]	A/Beijing/32/92 (H3N2)		A/Shandong/9/93 (H3N2)	
	aIIV3	IIV3‐sub1	aIIV3	IIV3‐sub1
	HI GMT (95% CI)		HI GMT (95% CI)	
	541 (416–703); *p* = 0.002	290 (219–384)	382 (280–522); *p* = 0.011	243 (181–326)
Del Giudice et al. [49]	A/Panama/1999 (H3N2)		A/Wyoming/3/2003 (H3N2)	
	aIIV3	IIV3‐sub1 and IIV3‐split1	aIIV3	IIV3‐sub1 and IIV3‐split1
	HI GMT (95% CI)		HI GMT (95% CI)	
	268.6 (208.8–345.5)	IIV3‐sub: 174.0 (118.3–255.9) IIV3‐split1: 141.2 (100.4–198.5)	181.0 (134.3–243.9); *p* = 0.0064 versus both	IIV3‐sub: 122.3 IIV3‐split1: 82.2
	HI seroprotection		HI seroprotection	
	98.3%	IIV3‐sub: 96.5% IIV3‐split1: 96.7%	98.3%; *p* = 0.0001 versus both	IIV3‐sub1: 75.9% IIV3‐split1: 80.0%
Ansaldi et al. [40]	aIIV3	IIV3‐sub1	aIIV3	IIV3‐sub1
	A/Wyoming/3/03 (H3N2)		A/Panama/2007/99 (H3N2)	
	HI GMT		HI GMT	
	347.7	129.9	422.3; *p* < 0.05	205.3
	HI seroconversion		HI seroconversion	
	68%; *p* < 0.05	28%	60%; *p* < 0.05	24%
			A/California/7/04 (H3N2)	
			HI GMT	
			50.5	21.9
			HI seroconversion	
			48%; *p* < 0.05	8%
			A/Wisconsin/67/05 (H3N2)	
			HI GMT	
			38.3; *p* < 0.05	23.2
			HI seroconversion	
			44%	20%
Ansaldi et al. [39]	Significantly higher postvaccination HI GMTs against four of six homologous strains with aIIV3 versus IIV3‐sub1 (see Figure 2)		Significantly higher postvaccination HI GMTs against eight of nine, and significantly higher seroconversion rates against five of nine, heterologous strains with aIIV3 versus IIV3‐sub1. Differences increased in magnitude with increasing genetic and antigenic distances between vaccine and drifted circulating strains (see Figure 2)	
Baldo et al. [50]	aIIV3	SVV and IIV3‐split2	aIIV3	SVV and IIV3‐split2
	A/H1N1/Beijing/262/95		A/H1N1/New Caledonia/20/99	
	GMT		GMT	
	~99; *p* < 0.05 versus SVV	SVV: ~80 IIV3‐split2: ~95	~98; *p* < 0.01 versus both	SVV: ~70 IIV3‐split2: ~60
	4‐fold increase in HI titers		4‐fold increase in HI titers	
	94.4%	SVV: 84.6% IIV3‐split2: 85.2%	68.1%; *p* < 0.05 versus both	SVV: 43.6% IIV3‐split2: 31.8%
	A/H3N2/Sydney/5/97		A/H3N2/Wisconsin/67/2005	
	GMT		GMT	
	~60; *p* < 0.01 versus both	SVV: ~48 IIV3‐split2: ~50	~50; *p* < 0.01 versus both	SVV: ~25 IIV3‐split2: ~30
	4‐fold increase in HI titers		4‐fold increase in HI titers	
	76.4%	SVV: 61.5% IIV3‐split2: 62.5%	41.7%; *p* < 0.05 versus both	SVV: 22.9% IIV3‐split2: 27.3%
	B/Beijing/184/93		B/Malaysia/2506/2004	
	GMT		GMT	
	~60; *p* < 0.05 versus both	SVV: ~42 IIV3‐split2: ~47	~20; *p* < 0.05 versus SVV	SVV: ~15 IIV3‐split2: ~18
	4‐fold increase in HI titers		4‐fold increase in HI titers	
	66.7%; *p* < 0.05 versus SVV	SVV: 46.2% IIV3‐split2: 54.5%	25.0%; *p* < 0.05 versus SVV	SVV: 7.7% IIV3‐split2: 26.1%
Song et al. [51]	A/Brisbane/59/2007 (H1N1)‐like strain, A/Brisbane/10/2007 (H3N2)‐like strain		A/California/7/2009 (H1N1), A/New Caledonia/20/1999 (H1N1), A/Solomon Island/3/2006 (H1N1), A/Wisconsin/67/2005 (H3N2)	
	No significant difference between aIIV3 and IIV3‐split3 in HI seroconversion or 1‐month postvaccination GMT values for any homologous or heterologous vaccine strain (*p* ≥ 0.05 for all comparisons)			
Camilloni et al. [52]	aIIV3	IIV3‐split4 (intradermal)	aIIV3	IIV3‐split4 (intradermal)
	HI GMT (95% CI)		HI GMT (95% CI)	
	A/Perth/16/09 (H3N2)		A/Perugia/06/12 (H3N2)	
	83.7 (44.8–156.2)	131.3 (72.3–238.5)	43.6 (30.8–61.8)	58.6 (41.1–83.5)
			A/Perugia/20/12	
			35.4 (23.3–53.3)	38.0 (26.6–54.3)
			A/Perugia/44/12 (H3N2)	
			50.1 (32.0–78.5)	56.6 (41.2–77.6)
			A/Perugia/50/12 (H3N2)	
			61.7 (42.1–90.3)	75.9 (50.6–113.9)
Frey et al. [32]	GMT ratio (aIIV3/IIV3‐sub2) (95% CI)		GMT ratio (aIIV3/IIV3‐sub2) (95% CI)	
	A/California/7/2009 (H1N1): 1.40 (1.32–1.49) A/Perth/16/2009 (H3N2): 1.61 (1.52–1.70) B/Brisbane/60/2008:1.15 (1.08–1.21)		A/Brisbane/10/2007 (H3N2): 1.45 (1.29–1.63); *p* < 0.001 A/Wisconsin/67/2005 (H3N2): 1.36 (1.23–1.50); *p* < 0.001 B/Malaysia/2506/2004:1.09 (0.98–1.21)	
	Seroconversion difference (aIIV3–IIV3‐sub2) (95% CI)		Seroconversion difference (aIIV3–IIV3‐sub2) (95% CI)	
	A/California/7/2009 (H1N1): 9.2 (7.1–11.3) A/Perth/16/2009 (H3N2): 12.7 (10.5–14.9) B/Brisbane/60/2008:5.2 (3.0–7.4)		A/Brisbane/10/2007 (H3N2): 11.3 (6.7–15.9); *p* < 0.001 A/Wisconsin/67/2005 (H3N2): 11.9 (7.3–16.6); *p* < 0.001 B/Malaysia/2506/2004:4.0 (−0.4 to 8.4)	
Dunning et al. [53]	HD‐IIV3	IIV3	HD‐IIV3	IIV3
	A/Victoria/361/2011 (H3N2)		A/Victoria/361/2011 (H3N2)^b^	
	HI GMT (95% CI)		HI GMT (95% CI)	
	459.97 (440.80–479.96)	252.78 (241.64–264.43)	49.60 (44.35–55.47)	33.59 (30.04–37.56)
	GMT ratio (HD‐IIV3/IIV3)		GMT ratio (HD‐IIV3/IIV3)	
	1.82 (95% CI, 1.71–1.94)		1.48 (95% CI, 1.26–1.73)	
	50% correlate of protection threshold: 203–437 80% correlate of protection threshold: ≥ 271		50% correlate of protection threshold: 22.4–44.7 80% correlate of protection threshold: 27.5–83.3	
Nicolay et al. [54] (meta‐analysis)	aIIV3	IIV3	aIIV3	IIV3
	24 strains: 7 A(H1N1), 10 A(H3N2), 7 B		10 strains: 2 A(H1N1), 6 A(H3N2), 2 B	
	GMT ratios (aIIV3/IIV3) and seroconversion differences (aIIV3–IIV3) statistically significant for all homologous strains		GMT ratios (aIIV3/IIV3): statistically significant for 7 of 10 strains: 2/2 A/H1N1; 4/6 A/H3N2; 1/2 B Seroconversion differences (aIIV3–IIV3): statistically significant for 9 of 10 strains: 2/2 A/H1N1; 6/6 A/H3N2; 1/2 B	
Belongia et al. [55]	RIV4	aIIV3 and HD‐IIV3	RIV4	aIIV3 and HD‐IIV3
	A/Hong Kong/4801/2014		A/Kansas/14/2017 (H3N2)	
	MN GMT (95% CI)		MN GMT (95% CI)	
	56.4 (39.3–81.1); *p* = 0.29^c^	aIIV3: 42.7 (25.8–70.6) HD‐IIV3: 53.2 (32.2–87.7)	48.5 (27.6–85.2); *p* = 0.06^c^	aIIV3: 22.7 (16.4–31.4) HD‐IIV3: 28.6 (17.4–47.0)
	MN seroconversion (95% CI)		MN seroconversion (95% CI)	
	13.3 (3.8–30.7); *p* = 0.49^c^	aIIV3: 6.7 (0.8–22.1) HD‐IIV3: 3.5 (0.09–17.8)	33.3% (95% CI, 16.5–50.2); *p* = 0.003^c^	aIIV3: 6.7% (95% CI, 0.8–22.1) HD‐IIV3: 3.5% (95% CI, 0.1–17.8)

Abbreviations: aIIV3, MF59‐adjuvanted trivalent influenza vaccine; GMT, geometric mean titer; HD‐IIV3, high‐dose trivalent influenza vaccine; Het, heterologous strain; HI, hemagglutination inhibition; Hom, homologous strain; IIV3, nonadjuvanted trivalent influenza vaccine; IIV3‐split1, nonadjuvanted split virion trivalent influenza vaccine; IIV3‐split2, nonadjuvanted split virion trivalent influenza vaccine; IIV3‐split3, nonadjuvanted split virion trivalent influenza vaccine; IIV3‐split4, nonadjuvanted split virion, intradermal trivalent influenza vaccine; IIV3‐sub1, nonadjuvanted subunit trivalent influenza vaccine; IIV3‐sub2, nonadjuvanted subunit trivalent influenza vaccine; IIV4, nonadjuvanted quadrivalent influenza vaccine; MN, microneutralization; Ref, reference strain; RIV4, quadrivalent recombinant influenza vaccine; SVV, trivalent subunit virosomal vaccine.

^a^ Vaccine being tested in the trial.

^b^ Seasonal mismatch due to egg adaptation of A/Victoria/361/2011 reference virus, not antigenic drift of circulating A/Victoria/361/2011.

^c^ *p* value for heterogeneity among all three comparators: RIV4, aIIV3, and HD‐IIV3.

### 3.1 Adjuvanted Influenza Vaccines

In most (but not all) comparisons, aIIV3 elicited more robust immune responses than non‐adjuvanted vaccines. The first randomized, controlled trial of the immunogenicity of enhanced influenza vaccines against heterologous strains examined antibody responses over three consecutive seasons from 1993 to 1995 in 437 subjects aged 64–87 years. The study compared aIIV3 to a nonadjuvanted, standard‐dose trivalent subunit influenza vaccine (IIV3; Agrippal, Novartis Vaccines, Basel, Switzerland). As measured with hemagglutination inhibition (HI) 28 days after vaccination, geometric mean titers (GMTs) against the three influenza vaccine antigens from A(H1N1), A(H3N2), and B strains were significantly higher in subjects vaccinated with aIIV3 versus IIV3, with the greatest differences observed in subjects with low pre‐existing immunity. aIIV3 also raised significantly higher neutralizing (NT) antibody titers against a drifted A(H3N2) strain, A/Shangdong/9/93, compared with IIV3 (Table 2) [48].

The immunogenicity of aIIV3 was compared with a nonadjuvanted split virion trivalent influenza vaccine (Begrivac; Novartis Vaccines, Basel, Switzerland) and a subunit IIV3 (Agrippal) in 119 older adults aged 61–91 years during the 2003–2004 season. The three vaccines elicited similar HI antibody responses to the homologous A(H3N2) strain, A/Panama/2007/1999. However, the predominant strain that season was A/Fujian/41112002. When tested against A/Wyoming/3/2003 (an A/Fujian‐like strain), aIIV3 induced seroprotective antibody levels (HI titer ≥ 40) in > 98% of individuals compared with 75.9% for the subunit IIV3 and 80% for the split virion IIV3 (Table 2) [49].

**FIGURE 2:**
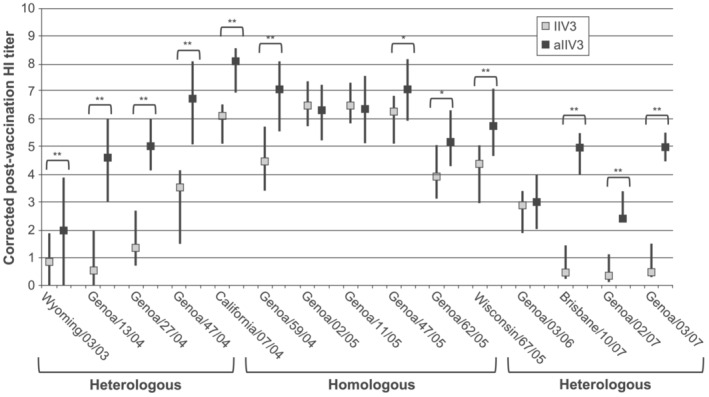
Postvaccination hemagglutination inhibition (HI) titers, corrected for prevaccination status, in subjects vaccinated with aIIV3 (MF59‐adjuvanted inactivated influenza vaccine) or nonadjuvanted IIV3 (nonadjuvanted inactivated influenza vaccine), each containing A/California/7/2004 antigen. Data expressed as median. Error bars denote 25%–75% interquartile range. *p < 0.05; **p < 0.01. Modified and reprinted with permission from Ansaldi et al. [39].

Two studies compared the impact of aIIV3 and a subunit IIV3 (Agrippal) on multiple drifted A(H3N2) strains (Table 2). In the first study, 50 healthy older adults aged ≥ 65 years were vaccinated with either aIIV3 or IIV3 during the 2004–2005 season. GMTs and seroconversion rates (percentage of subjects with HI titers ≥ 40) against the vaccine strain (A/Wyoming/3/03) were significantly higher among aIIV3 than IIV3 recipients (*p* < 0.05). Against three drifted A(H3N2) variants tested in the study, aIIV3 also induced significantly higher GMTs. These findings were confirmed by evaluation of NT titers (Table 2) [40]. In the second study, 50 adults older than 65 years received aIIV3 or IIV3 during the 2005–2006 season, and antibody responses were evaluated for 15 A(H3N2) strains belonging to different clades that circulated over the three seasons between 2004 and 2007. These included six homologous strains comprising A/California/7/04, the A(H3N2) used in the 2005–2006 seasonal influenza vaccine, and five antigenically similar strains. The other nine strains were heterologous and included A/Wyoming/3/03, the A(H3N2) strain used in the vaccine for the 2004–2005 season, in addition to three antigenically similar strains; four strains that fell within the clade represented by A/Brisbane/10/07; and A/Genoa/3/07, a A/Nepal/921/06‐like strain. As measured with HI or NT GMT ratios, subjects vaccinated with aIIV3 showed significantly higher postvaccination antibody responses than IIV3 recipients to four of six homologous and eight of nine drifted strains, including the drifted A/Brisbane/10/07‐like and A/Nepal/921/06‐like strains (Figure 2). In addition, HI titers and seroconversion rates were significantly higher among aIIV3 versus IIV3 recipients against the heterologous Wyoming/3/03‐like strains (Table 2). Differences between aIIV3 and IIV3 were not significant for A/California/7/04 and two homologous strains, but HI and NT titers in aIIV3 recipients were higher against the other three homologous strains, which each contained at least one amino acid change in an antigenic site. A correlation analysis showed that immunogenicity differences between the vaccines became even greater as the genetic and antigenic distances (calculated as the ratio between the HI titer of the reference serum with the reference strain and the HI titer of the reference serum with the clinical isolate [i.e., HI index]) between vaccine and drifted circulating strains increased. In summary, these direct comparisons showed that compared with the non‐adjuvanted vaccine, adjuvanted vaccine was associated with enhanced immunogenicity and a broader immune response against drifted strains [39].

Responses to heterologous virus strains were examined in a study comparing aIIV3 with split‐virion IIV3 (Mutagrip, Pasteur Merieux MSD, Lyon France) and a trivalent subunit virosomal vaccine (SVV; InflexalV, Swiss Serum and Vaccine Institute, Bern, Switzerland). In this study, sera from 199 adults aged ≥ 65 years with chronic medical conditions who resided in long‐term care facilities that were originally collected in a randomized, controlled trial during the 1998–1999 season were re‐tested for responses to influenza viruses included in the 2006–2007 seasonal influenza vaccine, that is, A/New Caledonia/20/99 (A/H1N1)‐like virus, A/Wisconsin/67/2005 (A/H3N2)‐like virus, and B/Malaysia/2506/2004‐like virus [50, 59]. GMT titers against all three 2006–2007 vaccine strains were significantly higher in sera from patients vaccinated with aIIV3 versus the comparators. The rates of 4‐fold increases in HI titers were significantly greater in the aIIV3 than the SVV group for the A/H1N1 and B viruses and greater versus both comparators for the A/H3N2 virus (Table 2) [50].

In a study conducted in Korea during the 2009–2010 season, cross‐reactive immunogenicity was compared in adults aged ≥ 65 years randomized to aIIV3 (*n* = 47) and split‐virion IIV3 (*n* = 48; GCFlu, Green Cross, Seoul, Korea). The heterologous strains included pandemic influenza A/California/7/2009 (H1N1), A/New Caledonia/20/1999 (H1N1), A/Solomon Island/3/2006 (H1N1), and A/Wisconsin/67/2005 (H3N2). The authors found no significant differences between aIIV3 and IIV3 in evaluations of seroconversion or postvaccination HI GMT values for any of the heterologous vaccine strains (Table 2). aIIV3 did demonstrate more durable immunogenicity against the homologous A/H3N2 virus, with significantly greater seroconversion rates in the aIIV3 than the IIV3 group [51].

During the 2011–2012 season, a study involving Italian nursing home residents aged ≥ 64 years (mean age, 85 years) evaluated responses to four A/H3N2 variants (A/Perugia/06/12, A/Perugia/20/12, A/Perugia/44/12, and A/Perugia/50/12) with aIIV3 and a split virion IIV3 formulation designed to be injected intradermally instead of intramuscularly (Intanza, Sanofi‐Pasteur, Lyon, France) [52]. This method of administration was associated with enhanced immune responses in previous studies [67, 68]. Among nursing home residents, HI GMT responses against A/Perugia/06/12 were significantly greater with the intradermal IIV3 than aIIV3. No other significant differences were found in the study (Table 2) [52]. The manufacturer ceased production of intradermal IIV3 after the 2017–2018 season [69].

In a large, phase III, randomized, observer‐blinded, multicenter study conducted during the 2010–2011 season, the immunogenicity of aIIV3 versus a subunit IIV3 (Agriflu, Novartis Vaccines, Basel, Switzerland) was evaluated in 7082 adults ≥ 65 years of age. Postvaccination HI antibody responses were significantly greater with aIIV3 versus IIV3 against all homologous strains—A(H3N2) in particular—as well as against two heterologous A(H3N2) strains (*p* < 0.001) but not a heterologous B strain. Results were similar in subjects with comorbidities putting them at high risk from influenza (Table 2) [32].

A recent meta‐analysis of 23 aIIV3 trials conducted between 1992 and 2013 included four trials with heterologous strain data. In these studies, HI titers were significantly greater with aIIV3 versus IIV3 for 7 of 10 strains, including 2 of 2 A(H1N1), 4 of 6 A(H3N2), and 1 of 2 B strains. Significantly higher seroconversion rates were seen for all strains except 1 B strain comparison (Table 2) [54].

### 3.2 Nonadjuvanted High‐Dose Influenza Vaccine

Only one study has examined the immunogenicity of HD‐IIV3 against a mismatched strain. In 675 adults aged ≥ 65 years who participated in a large phase III trial of HD‐IIV3 (*N* = 31,989), blood samples were evaluated for immunogenicity and correlates of protection [45, 53]. The main trial was conducted over two seasons (2011–2012 and 2012–2013), of which the second season was mismatched due to egg adaptation of the A(H3N2) strain used in the vaccine (A/Victoria/361/2011), whereas the circulating A/Victoria/361/2011 remained antigenically similar to the cell‐propagated reference virus. Compared with IIV3, HD‐IIV3 induced significantly higher HI titers against both the vaccine and circulating A/Victoria strains (Table 2). Due to season mismatch, the HI titers required to meet 50% correlate of protection thresholds were higher for the vaccine (203–437, depending on case definition) than circulating virus (22.4–44.7). Titers induced by HD‐IIV3 (but not IIV3) met the 50% protection threshold for both the vaccine and circulating strains for all six case definitions used in the study. However, at the 80% protection level, HD‐IIV3‐induced titers against the vaccine strain did not meet the 80% protection threshold for four of six case definitions for the vaccine (HI titer ≥ 1096) and circulating (HI titer 51.0–83.3) strains (Table 2) [53].

### 3.3 Nonadjuvanted Recombinant Influenza Vaccine

Only one study has evaluated the immunogenicity of the higher than standard dose RIV4 against a heterologous virus. In a planned substudy of a trial that evaluated serological responses to sequential vaccination with aIIV3, HD‐IIV3, and IIV3 in adults aged 65–74 years over two influenza seasons (2016–2017 and 2017–2018), 60 participants who received IIV3 during the first season, as well as 29 newly recruited subjects (total *N* = 89), were randomized to receive aIIV3, HD‐IIV3, or RIV4 during the second season. In addition, immune responses to an antigenically drifted A(H3N2) variant were evaluated in the substudy, whereas the main trial did not evaluate responses to heterologous viruses [55, 70]. Postvaccination microneutralization (MN) GMTs, MN titers ≥ 1:40, and seroconversion rates (defined as > 4‐fold rise in MN titer with post vaccination titer > 1:40) against three homologous A(H3N2) strains were not statistically different between the three vaccine groups, and GMT and titers ≥ 1:40 against the drifted variant, A/Kansas/14/2017, also did not differ statistically among the three vaccines (*p* = 0.06). Rates of seroconversion against A/Kansas were higher in the RIV4 group than the aIIV3 or HD‐IIV3 groups, however (Table 2). Seroconversion rates were also higher among RIV4 recipients in the cohort that was seronegative (MN titer < 1:10) at baseline [55].

## 4 Discussion

Antigenic drift is a key driver of influenza virus evasion of the human immune system and the need to reformulate influenza vaccines each season. Given the vulnerability of older adults to influenza complications, vaccines that can provide cross‐protection meet an important need. However, few studies have compared cross‐protection with enhanced versus standard influenza vaccines, and even fewer have compared cross‐protection among enhanced vaccines.

Nevertheless, available data support the use of enhanced vaccines for older adults during mismatched influenza seasons [32, 39, 40, 48, 49, 53, 54, 55]. Compared with nonadjuvanted, standard‐dose influenza vaccines, both adjuvanted and high‐dose influenza vaccines elicited significantly higher antibody titers in separate studies (Table 2). More studies have been conducted with aIIV3 than with other enhanced vaccines, and studies of aIIV3 consistently demonstrate broad cross‐reactivity against heterologous strains, including when tested against nine different A(H3N2) variants [32, 39, 40, 48, 49, 54]. A study of HD‐IIV3 versus IIV3 demonstrated that a higher immune response is correlated with improved protection from clinical outcomes, although in this study the high‐dose vaccine failed to provide 80% protection from four of six clinical outcomes [53].

The mechanism by which aIIV enhances immune responses differs from that of HD‐IIV and RIV4. The latter two enhanced vaccines increase the magnitude of the immune response with higher doses of antigen (60 μg HA per strain in HD‐IIV and 45 μg HA per strain in RIV4, compared with 15 μg HA per strain in standard dose influenza vaccines) [29]. The MF59 adjuvant in aIIV3 has an antigen‐sparing effect, which permits this vaccine to increase the magnitude of the immune response with a standard dose of antigen (15 μg HA per strain) while also extending the breadth of the immune response by enhancing production of cross‐reactive antibodies [30, 71, 72, 73]. Because of the ability to promote a cross‐reactive immune response, MF59‐adjuvanted vaccines may be used for immunization against pandemic influenza strains as well as seasonal influenza [72].

To date, only one study has explored heterologous immune responses among enhanced vaccines, comparing a quadrivalent recombinant vaccine with trivalent vaccines (Table 2). In the 2017–2018 influenza season, MN seroconversion rates against a heterologous virus were higher with RIV4 versus aIIV3 or HD‐IIV3, there were no significant differences in MN GMTs for that strain, and no significant differences in any measure for the homologous strains [55]. Whether any of these three vaccines offers benefits beyond the others has not yet been determined [9].

This review has its limitations. Only published trials searchable in the US National Library of Medicine database were obtained, and because few trials of HD‐IIV and RIV4 have been published, results for those vaccines were limited. Trials differed in demographic characteristics of the subjects and the years conducted. The numbers of subjects in specific trials varied, and comparisons were limited to the enhanced vaccines studied. Nonetheless, this review is the first such analysis of existing RCT data to date on an important public health question of the role of enhanced influenza vaccine protection against drifted strains.

The paucity of heterologous immunogenicity data for enhanced vaccines, especially the more recently licensed HD‐IIV and RIV4, is an important gap in our understanding of vaccine efficacy and effectiveness, especially in mismatched seasons. Given the labile nature of A(H3N2), which is susceptible to not only antigenic drift but egg adaptation, which both may lead to mismatch between vaccine and circulating strains, further work in this area is warranted.

## Author Contributions

**John Youhanna:** Conceptualization; Funding acquisition; Investigation; Methodology; Resources; Supervision; Writing – original draft. **Vy Tran:** Conceptualization; Investigation; Methodology; Resources; Writing – original draft. **Randall Hyer:** Investigation; Writing – review and editing. **Alexander Domnich:** Investigation; Writing – review and editing.

## Conflicts of Interest

JY and VT are employed by CSL Seqirus Ltd. RH and AD report receiving funding for investigational work from CSL Seqirus Ltd.

## Data Availability

Data availability is not applicable to this article as no new data were created or analyzed in this study.
